# From Metabolic Syndrome to Atrial Fibrillation: Linking Inflammatory and Fibrotic Biomarkers with Atrial Remodeling and Imaging-Based Evaluation—A Narrative Review

**DOI:** 10.3390/metabo16010059

**Published:** 2026-01-09

**Authors:** Adrian-Grigore Merce, Daniel-Dumitru Nisulescu, Anca Hermenean, Oana-Maria Burciu, Iulia-Raluca Munteanu, Adrian-Petru Merce, Daniel-Miron Brie, Cristian Mornos

**Affiliations:** 1 Doctoral School Medicine–Pharmacy, “Victor Babeș” University of Medicine and Pharmacy Timișoara, E. Murgu Sq. No. 2, 300041 Timișoara, Romania; adrian-grigore.merce@umft.ro (A.-G.M.);; 2 Institute of Cardiovascular Diseases Timisoara, 300310 Timisoara, Romania; 3 Advanced Research Center, Institute for Cardiovascular Diseases of Timișoara, 300310 Timișoara, Romania; 4 Multidisciplinary Doctoral School, Vasile Goldiș Western University of Arad, 310025 Arad, Romania; 5 Department of Histology, Faculty of Medicine, Vasile Goldiș Western University of Arad, 310025 Arad, Romania; 6 Aurel Ardelean Institute of Life Sciences, Vasile Goldiș Western University of Arad, 310025 Arad, Romania; 7 Department of Cardiovascular Surgery, Institute of Cardiovascular Diseases, 300310 Timișoara, Romania; 8 Cardiology Department, “Victor Babeș” University of Medicine and Pharmacy, 2 Eftimie Murgu Sq., 300041 Timișoara, Romania

**Keywords:** atrial fibrillation, atrial fibrosis, metabolic syndrome, inflammatory biomarkers, TGF-β1, TNF-α, IL-6, galectin-3, galectin-1, atrial remodeling

## Abstract

Atrial fibrillation (AF) is the most prevalent sustained arrhythmia worldwide and is now increasingly regarded as a disease of chronic inflammation and progressive atrial fibrosis. Understanding of molecular mechanisms that mediate the linkage between systemic metabolic dysregulation, inflammation, and structural atrial changes is crucial for informing risk stratification and targeting of prevention strategies. This review provides evidence from 105 studies focusing on the contributions of transforming growth factor-β1 (TGF-β1), tumor necrosis factor-a (TNF-α), interleukin-6 (IL-6), galectin-3, and galectin-1 to cardiac fibrogenesis, atrial fibrosis, and AF pathogenesis. We also link metabolic syndrome to these biomarkers and to atrial remodeling, as well as echocardiographic correlates of fibrosis. TGF-β1 is established as the central profibrotic cytokine and promotes Smad-based fibroblast activation, collagen accumulation, and structural atrial remodeling. Its role is highly potentiated by thrombospondin-1 by turning latent TGF-β1 into its potent form. TNF-α and IL-6 also play an integral role in the inflammatory fibrotic continuum by activating NF-κB and STAT3 signaling, promoting fibroblast proliferation, electrical uncoupling, and extracellular matrix accumulation. Galectin-3 is a potent profibrotic mediator that promotes TGF-β signaling and is a risk factor for negative outcomes, whereas Gal-1 seems to regulate inflammation resolution and may exert context-dependent protective or maladaptive roles. Metabolic syndrome is strongly associated with excessive levels of these biomarkers, chronic low-grade inflammation, oxidative stress, and ventricular and atrial fibrosis. Chronic clinical findings show that metabolic syndrome (MetS) increases AF risk, exacerbates atrial dilatation, and is associated with worse postoperative outcomes. Echocardiographic data are connected to circulating biomarkers and are non-invasive for evaluating atrial remodeling. The evidence to date supports that atrial fibrosis should be considered an end point of systemic inflammation, metabolic dysfunction, and activation of profibrotic molecular pathways. Metabolic syndrome, due to its chronic low-grade inflammatory environment and prolonged levels of metabolic stress, manifests as an important upstream factor of fibrotic remodeling, which continuously promotes the release of cytokines, oxidative stress, and fibroblast activation. Circulating fibrotic biomarkers, in comparison with metabolic syndrome, serve separate yet interdependent pathways that help orchestrate atrial structural remodeling through the simultaneous process but can also provide a long-term indirect measure of ongoing profibrotic activity. The integration of these biomarkers with superior atrial imaging enables a broader understanding of the fibrotic substrate of atrial fibrillation. This combined molecular imaging approach can facilitate risk stratification, refine therapeutic decisions, and facilitate early identification of higher-risk metabolic phenotypes, thus potentially facilitating directed antifibrotic and anti-inflammatory therapy in atrial fibrillation.

## 1. Introduction

Atrial fibrillation (AF) is the most prevalent arrhythmia in health-related arrhythmias and is characterized by disjointed atrial electrical activity and uncoordinated contractions of the atria. Worldwide, about 0.5% of the world population has AF; approximately 37 million patients accounted for around 37 million cases in 2017 [1]. However, growing population aging has continued to drive a steadily increasing prevalence worldwide. More than 11 million people in Europe are estimated to have AF [2], and the disease is expected to double its incidence by 2060. AF has thus become a genuine global epidemic, posing one of the most important public health challenges [3]. The clinical and socioeconomic effects of AF are great. AF is a leading risk factor for ischemic stroke, representing ~20–30% of all strokes, and raises the risk of HF by five-fold. Without adequate anticoagulants, people with AF have an average 2–5-fold increased risk of stroke compared with those in sinus rhythm [4]. AF is also linked to a twofold increase in cardiovascular mortality, a substantial decrease in quality of life and symptoms, as well as high rates of hospitalizations due to AF [5]. Economically speaking, it is a weight, as we can see from the cost to healthcare systems: AF management takes up to 2.6% of the annual healthcare bill in European countries. This effect is primarily related to hospitalizations and thromboembolic damage and the financial burden on patients in the context of AF-related stroke, which amounts to tens of billions of euros annually [6,7,8]. From a pathophysiologic perspective, both the onset and development of AF share an association with structural atrial remodeling (AF), and one of such mechanisms is the formation of atrial fibrosis, owing to the factors of hemodynamic overload, chronic inflammation, and oxidative stress. Atrial fibrosis causatively contributes to arrhythmogenesis as it causes discontinuous conduction and re-entry, but it is also a consequence of AF. It has been demonstrated that atrial fibrosis can be a cause and an effect of AF, and when established, it acts as a factor in the evolution of the arrhythmia and a continuum of atrial fibrillation resulting in atrial fibrosis (“atrial fibrosis develops AF, and AF creates fibrosis”) [9]. The degree of atrial fibrosis correlates with the clinical course of AF and with the efficacy of rhythm control strategies. Thus, atrial fibrosis represents a significant arrhythmogenic substrate driving AF persistence and recurrence after treatment. Within this framework, a great deal of effort is being put forward to identify molecular biomarkers that characterize atrial fibrotic burden and therefore are compatible with non-invasive imaging methods, biomarkers that are also linked with metabolic syndrome and insulin resistance. The molecules TGF-β1, TNF-α, IL-6, galectin-3, and galectin-1 are critically involved in the cardiac inflammatory and profibrotic cascade, and they have been linked to myocardial fibrosis severity and to structural and electrical atrial remodeling. We selected these five biomarkers due to their well-established roles in atrial inflammation and remodeling. And as we present in the review article, transforming growth factor β1 (TGF-β1) is a one of the main pro-fibrotic cytokine in atrial remodeling; tumor necrosis factor α (TNF-α) and interleukin 6 (IL-6) are central pro-inflammatory mediators that are elevated in metabolic syndrome and have been linked to AF pathogenesis; galectin-3 (Gal-3) is a lectin that promotes atrial fibrosis and is predictive of AF and galectin-1 (Gal-1) similarly regulates immune responses and has emerging relevance in cardiac tissue remodeling (Table 1).

Metabolic rewiring accompanies fibrotic signaling in the heart. High-throughput metabolomics has revealed that cardiac fibroblast activation driven by TGF-β1 and related cues involves major shifts in energy metabolism. Reviews note that failing fibrotic hearts show these patterns accompanying fibrosis. In an integrative proteomics–metabolomics study of cardiac resynchronization therapy, re-wiring of metabolism was directly linked to TGF-β1 signaling: overexpressing the metabolic regulator 4EBP1 suppressed TGF-β1-induced fibroblast proliferation and collagen production [10,11].

Galectins and fibrosis leave metabolic fingerprints. Gal-3 and Gal-1 correlate strongly with lipid and inflammatory metabolites. In a large human cohort, plasma Gal-1 and Gal-3 levels were each associated with numerous fatty acids, lipoproteins, and triglyceride-related metabolites in untargeted metabolomic profiling. In heart failure patients, a composite “metabolite score” tracked with fibrosis: patients with higher metabolomic scores had higher Gal-3 and worse outcomes, and the metabolite score outperformed Gal-3 in predicting decompensation [12,13].

**Figure 1:**
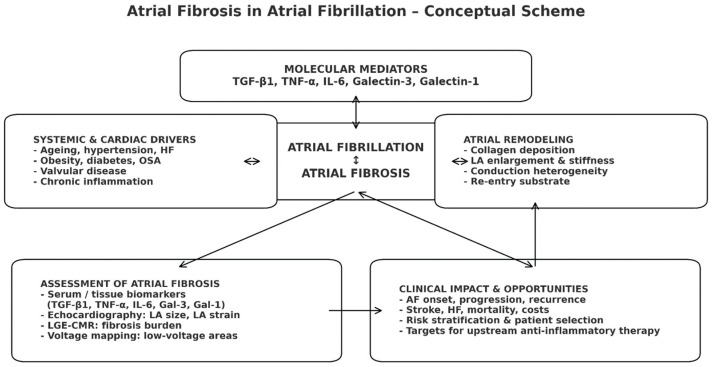
Conceptual scheme of atrial fibrosis in atrial fibrillation. Systemic and cardiac drivers activate profibrotic molecular mediators, promoting atrial fibrosis that is mutually reinforcing with atrial fibrillation. Resulting structural remodeling leads to adverse clinical outcomes, while imaging and biomarker assessment of fibrosis support risk stratification and selection for upstream anti-inflammatory/antifibrotic therapies.

Inflammatory cytokines imprint on the metabolome. AF and cardiac fibrosis are also driven by inflammation. Patients with heart failure with preserved ejection fraction exhibit elevated tryptophan–kynurenine metabolites, a signature catabolite of inflammatory activity. Serum kynurenine and indoleacetate were significantly higher in patients versus controls [14]. In several studies, their serum or tissue fractions have been correlated with atrial size and functional parameters [15,16,17,18]. Combining these biomarkers with non-invasive methods to assess atrial fibrosis may provide greater refinement in risk stratification, predict response to ablation/cardioversion, and ultimately aid in the identification of anti-inflammatory and antifibrotic therapeutic targets (Figure 1). This review will attempt to synthesize the existing data, examining the potential involvement of these molecules in cardiac and atrial fibrosis and the associated links among serum/tissue biomarkers, metabolic syndrome, and echocardiographic biomarkers of atrial remodeling. By positioning these biomarkers at the crossover between metabolic syndrome–driven fibrosis and standard echocardiographic imaging, we aim to highlight their feasibility as markers of atrial fibrosis displaying important diagnostic and prognostic potential, providing a basis for future therapeutic decisions and individualized treatment strategies involving atrial fibrillation. A key potential benefit of identifying these markers and associating them with AF diagnosis is their applicability as AF screening modalities in patients with pre-existing risk factors, at the same time as they hold pre-existing risk factors that are not yet fully accepted as conventional risk factors, together to lessen the devastating effects of cardioembolic adverse events of atrial fibrillation.

## 2. Materials and Methods

**Figure 2:**
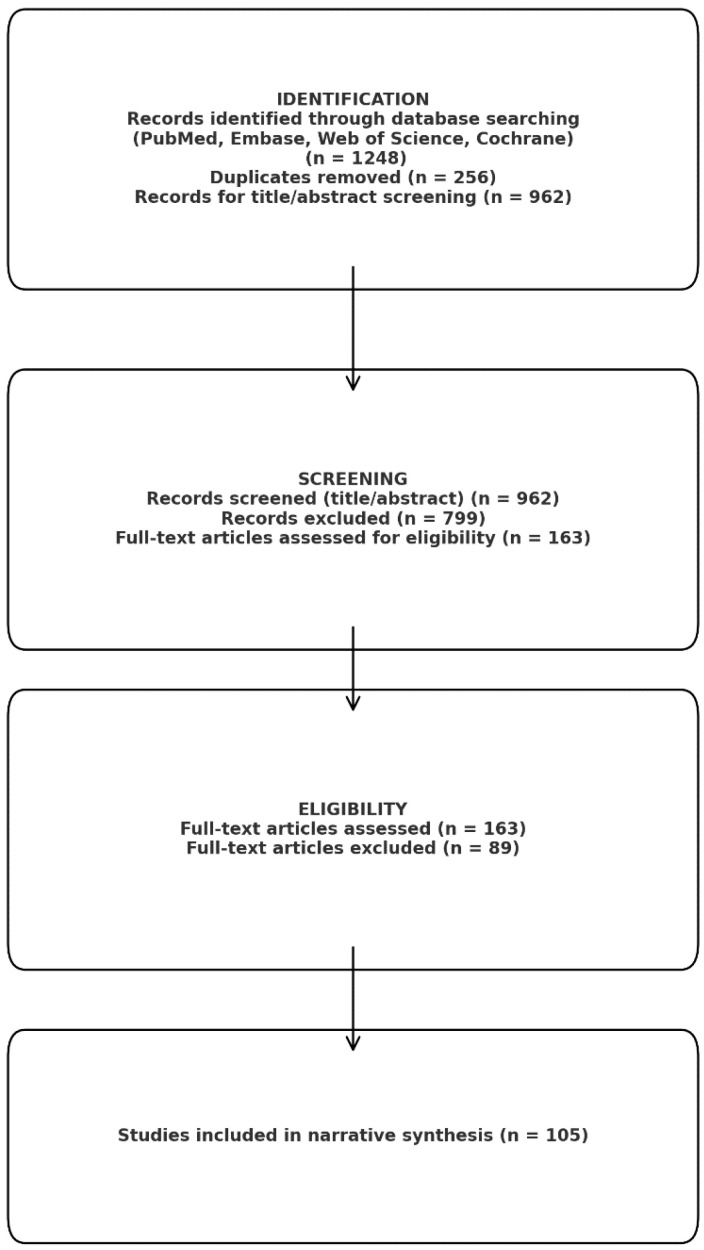
Prisma analysis.

We performed a narrative review in a systematic literature search attempt to synthesize information about the contribution of the key profibrotic and pro-inflammatory molecules (TGF-β1, TNF-α, IL-6, galectin-3, and galectin-1) to cardiac fibrogenesis generally and in the atrial fibrosis associated with atrial fibrillation (AF) in particular. The goals were (1) to shed light on the molecular and cellular mechanisms that these mediators induce in cardiac structural remodeling, (2) to establish associations between serum or tissue concentrations of these mediators and the degree of atrial fibrosis, (3) to report interconnections between the biomarkers and atrial echocardiographic markers, and then to examine the prognosis of AF patients. The literature search utilized PubMed/MEDLINE, Embase, Web of Science, and the Cochrane Library from database inception to the date of last querying (September 2025). No lower time limit was applied to collect previous mechanistic observations and recent clinical studies. Search terms included (but were not limited to): “atrial fibrillation” OR “atrial arrhythmia” AND (“atrial fibrosis” OR “cardiac fibrosis” OR “atrial remodeling”) AND (“transforming growth factor beta” OR “TGF-β1” OR “tumor necrosis factor alpha” OR “TNF-α” OR “interleukin-6” OR “IL-6” OR “galectin-3” OR “galectin-1”). Further searches were made on the association of metabolic syndrome with cardiac fibrosis and on associations between metabolic syndrome and all of the molecular biomarkers tested. Search terms were modified for each database. Further, we included target terms for methods of fibrosis assessment (e.g., “late gadolinium enhancement”, “voltage mapping”, “speckle-tracking echocardiography”, “left atrial strain”), and the reference lists of relevant papers were compiled manually to determine sources found in further publications. After identification of 286 duplicates, a total of 962 articles were retained from the 1248 records and screened according to titles and abstracts of the articles. The 163 articles were then analyzed in full text, of which 105 studies were found to fit the inclusion criteria and included in the narrative synthesis (Figure 2). Figure 2 was created with the support of a generative artificial intelligence tool (Gemini *NanoBananaPro*) to visually synthesize and schematically integrate the molecular mechanisms described in the literature linking metabolic syndrome to atrial remodeling and atrial fibrillation. The scientific content (selection of pathways/biomarkers, directionality of interactions, and causal relationships) was defined by the authors based on the studies cited in the manuscript, and the figure was subsequently reviewed, edited, and finalized by the authors to ensure accuracy, coherence, and consistency with the narrative of the review.

## 3. Results

### 3.1. Methods for Assessing Atrial Fibrosis in Current Clinical Practice

Delayed-Enhancement MRI Determinant of Successful Radiofrequency Catheter Ablation of Atrial Fibrillation (DECAAF) has evidenced that there is a direct relationship between the degree of atrial fibrosis experienced and the risk of AF recurrence following treatment: each 1% increase in fibrosis detected in AF patients was correlated with a 6% increased risk of AF recurrence post ablation [19]. Currently available approaches for estimating fibrosis have major limitations, mainly due to the lack of possibility of accurately measuring it. LGE-MRI imaging of atrial fibrosis directly provides good visualizations of fibrotic tissue but is still less accurate and reproducible. No tight and consistent correlation of late gadolinium enhancement or low-voltage areas on electroanatomical mapping with true histological fibrosis. Indeed, atrial LGE-MRI is also characterized by very poor spatial resolution due to the fact that the atrial wall is much thinner than the ventricular wall and is not widely available [20]. Scarred low-voltage sites found on intracardiac electroanatomical maps may represent fibrosis that needs to be ablated and mapped with an invasive approach and are affected by many technical factors [9]. Atrial biopsy gives a direct diagnosis of fibrosis, but it is infrequently taken because of the invasiveness and associated dangers [21]. Therefore, in clinical practice, it is rare to see any reliable tools to quantify atrial fibrosis without using invasive means and to estimate the “fibrotic burden” in AF patients without the use of any instrument. Transthoracic echocardiography (TTE) is presently considered the main source of measurement to determine the size and function of the atrial fibrillation (AF) atrial atrioforma in clinical practice, as measures like left atrial diameter and volume that are derived from it will be left atrial diameter and volume but provide indices of the chronic remodeling. Increases in these parameters are suggestive of chronic dilatation of the atria, consistent with the duration and type of AF and correlating with duration and type [22,23]. Previous research has reported a significantly increased size of atria in AF compared to sinus rhythm patients [24]. The significance of the presence of the arrhythmia and the existence and endurance of the arrhythmia is emphasized, as the fibrillating atrial rhythm will, in and of itself, contribute to atrial enlargement. So the understanding of “AF begets AF” is strengthened: the arrhythmia induces chronic atrial dilatation that promotes its maintenance [25]. The standard echocardiographic metrics cannot correlate with myocardial fibrosis. The right atrial (RA) remodeling in the context of AF is less well characterized than the left atrial (LA) remodeling [26]. However, newer data suggest right atrial changes have prognostic significance as well. Independent of other risk factors, right atrial dilatation, expressed as indexed right atrial volume, has been shown to predict events with regard to incident AF. Patients who developed AF in the population-based MESA cohort were able to report considerably greater RA volumes at baseline than those without AF did. Adjusted for the conventional risk factors and LA parameters, these RA changes still remained prognostically significant: higher right atrial volume was independently correlated with an increased risk of de novo AF [27]. This demonstrates that both atria are involved in AF pathogenesis, and as a reservoir for systemic venous return, the right atrium is likely to dilate and lose function over the course of AF, as does the left atrium [28]. In addition, increased RA volume was reported to even outnumber LA volume in a clinical context as a predictor of the recurrence of arrhythmia following cardioversion or ablation and has been implicated as a risk factor of stroke in patients with AF [26]. Besides chamber size, functional atrial remodeling in AF appears to be demonstrated by defects in atrial pump and reservoir functioning. In the sinus rhythm, the left atrium represents a reservoir and passive conduit in early diastole but an active pump in late diastole. In AF, organized atrial contraction is bypassed, thus eliminating the booster-pump function and decreasing atrial compliance by means of atrial compliance impairment; therefore, there is a reduction in the function of the atria at rest. Modern speckle-tracking echocardiography offers parameters, such as left atrial strain, that quantify these functions. Left atrial reservoir strain (LASr) is significantly less in patients with AF than in patients in sinus rhythm, indicating both loss of contractile function and increased stiffness of the atrial wall secondary to fibrosis. In a study of atrial strain in AF patients, LASr was significantly lower in AF patients with atrial fibrosis compared with controls, and lower LASr was associated with extensive fibrosis seen by collagen gene expression in atrial appendage biopsies; AF patients exhibited higher type I collagen expression with a negative correlation between strain value and myocardial collagen density [29]. The main benefit of echocardiography is its wide availability and non-invasive method of atrial remodeling screening, which is attractive for monitoring. In the future, integration of these straightforward imaging markers with the serum biomarkers reported earlier would offer a composite estimate of atrial fibrosis without the necessity for expensive or invasive studies.

### 3.2. The Metabolic Syndrome—A Systemic Pro-Inflammatory Condition

**Figure 3:**
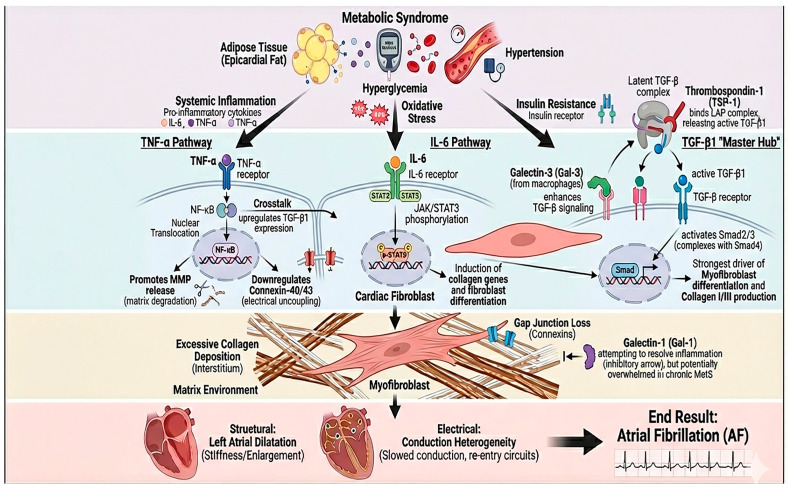
Inflammatory–fibrotic axis linking metabolic syndrome to atrial remodeling and atrial fibrillation. Components of metabolic syndrome (epicardial adipose tissue expansion, hyperglycemia/oxidative stress, hypertension, and insulin resistance) promote systemic inflammation, characterized by increased TNF-α and IL-6 signaling. TNF-α activates NF-κB with nuclear translocation, facilitating matrix remodeling (e.g., MMP release), downregulation of connexins (Cx40/Cx43), and electrical uncoupling, while also upregulating TGF-β1 through inflammatory–fibrotic crosstalk. In parallel, IL-6 signaling via the IL-6 receptor triggers JAK/STAT3 phosphorylation in cardiac fibroblasts, inducing profibrotic gene programs, collagen synthesis, and fibroblast differentiation. TGF-β1 acts as a central “master hub”: thrombospondin-1 (TSP-1) activates latent TGF-β, and macrophage-derived galectin-3 further amplifies TGF-β/Smad signaling, driving myofibroblast transformation and collagen I/III deposition. Although galectin-1 may counterbalance inflammation and fibroblast activation, this protective pathway may be insufficient in chronic metabolic stress. The net result is excessive interstitial fibrosis, gap-junction loss, left atrial dilation/stiffness, and conduction heterogeneity that culminate in atrial fibrillation.

Metabolic syndrome (MetS) is a heterogeneous entity of interconnected risk factors—central obesity, hyperglycemia/insulin resistance, atherogenic dyslipidemia, and hypertension—which induces a state of chronic systemic low-grade inflammation. The pathophysiological pathways by which MetS elicits inflammation and cardiac fibrogenesis are complex and multifactorial, with contributions from oxidative stress, insulin resistance, and pro-inflammatory cytokines. These factors activate profibrotic cellular pathways and promote negative cardiac remodeling. These mechanisms are described below, along with the clinical evidence concerning myocardial fibrogenesis and its relation to atrial fibrillation (AF) (Figure 3) [30,31,32].

#### 3.2.1. Metabolic Syndrome and the Fibrosis of the Heart

Cardiac fibrosis is the excessive accumulation of connective tissue within the myocardium, resulting in stiffening of the cardiac walls, impaired electrical conduction, and pump dysfunction. Fibrogenesis, at the ventricular level, is initially a reparative response following injury, but its excessive or chronic nature leads to adverse remodeling and heart failure. The fibrotic process is orchestrated by a complex interplay between the cells and the surrounding tissue: cardiac fibroblasts respond to profibrotic factors, including cytokines, hormones, and mechanical stress, and differentiate into myofibroblasts that proliferate and deposit collagen in the interstitial space. Thus, several signaling pathways—TGF-β, inflammatory mediators such as TNF-α, IL-6, angiotensin II, and oxidative stress—cooperate to activate fibroblasts, disrupt the balance between synthesis and degradation of extracellular matrix, and give rise to scar-like fibrotic tissue (Figure 3) [33,34,35,36,37,38,39,40,41,42,43].

##### Transforming Growth Factor β1

TGF-β1 is defined as the “master regulator” of cardiac fibrogenesis. This pleiotropic cytokine, secreted primarily by activated macrophages, fibroblasts, and stressed cardiomyocytes, activates the Smad signaling cascade, which induces the switch of fibroblasts into myofibroblasts and collagen deposition in the myocardium [44]. After cardiac injury, TGF-β1 levels rise, causing fibroblasts to proliferate and deposit collagen. This leads to scar formation that stiffens the ventricles and impairs function [45]. TGF-β1 in the heart originates primarily from infiltrating macrophages as well as resident fibroblasts, endothelial cells, and smooth muscle cells, which release TGF-β1 to respond to inflammatory or hormonal stimuli [46]. TGF-β1 binds to fibroblast receptors and activates Smad3/4, which increases collagen production and reduces collagen degradation. Towards this end, thrombospondin-1 (TSP-1) is emerging as an essential cofactor of the TGF-β1 pathway and plays a major role in activating the latent form of TGF-β stored in the extracellular matrix. TSP-1 activates latent TGF-β1, thereby amplifying its profibrotic signaling [47]. TSP-1 levels rise after pressure overload or infarction, and this correlates with higher collagen and ventricular stiffening [48,49,50]. Furthermore, TGF-β and other pathways have been shown to interact through crosstalk with one another; for instance, angiotensin II–endothelin-1 receptor activation increases local TGF-β expression and exaggerates fibrogenesis [51]. In experimental models, TGF-β signaling blockade markedly alleviates post-infarction fibrosis and enhances cardiac function, consistent with the pharmacological targets of TGF-β1, which appears as an effective target to prevent maladaptive remodeling [52]. In addition, progressive fibrosis in diabetic and hypertensive cardiomyopathy is largely facilitated by TGF-β1–Smad3 signaling [53]. In summary, TGF-β1 drives fibroblast activation and collagen deposition, and TSP-1 amplifies this profibrotic response. Elevated TGF-β1 is therefore linked to more intense fibrosis and dysfunction [54]. In this respect, it is not unexpected that patients suffering from advanced heart failure have high plasma TGF-β1 levels, which correlate with markers of negative remodeling and unfavorable prognosis [55].

##### Tumor Necrosis Factor α

TNF-α is a pro-inflammatory cytokine that links inflammation to cardiac remodeling. TNF-α is expressed primarily by activated macrophages in injured or stressed myocardium but also by cardiomyocytes, fibroblasts, and endothelial cells under appropriate stimuli, including oxidative stress [56]. Epicardial fat secretes TNF-α that diffuses into the myocardium and promotes local fibrosis [57]. Its profibrotic effects are mediated by several pathways. TNF-α directly activates cardiac fibroblasts, causing them to proliferate and produce collagen. It also stimulates TGF-β1 expression, reinforcing fibrosis. TNF can induce TGF-β1 expression in the heart and thus promotes the fibrotic cascade [58]. In addition, TNF-α stimulates the expression of the NF-κB transcription factor in myocardial cells and triggers an inflammation that can persist, leading to an elevated secretion of MMPs degrading the extracellular matrix [57]. TNF-α also down-regulates connexins (Cx43/Cx40), disrupting electrical coupling in the heart [59]. Patients with congestive heart failure often show elevated circulating TNF-α, which is associated with increasingly severe myocardial dilatation and fibrosis, and have a worse prognosis due to having more advanced diseases [60]. In animals, suppression of the TNF-α/NF-κB cascade was able to decrease fibrosis, post-infarction, and remodeling with a better outcome [61,62].

##### Interleukin-6

IL-6 is a cytokine. In the heart, IL-6 is produced by a wide range of cells [63]. IL-6 activates the JAK/STAT3 pathway in fibroblasts, inducing genes for collagen production and myofibroblast differentiation. Thus, the IL-6/STAT3 cascade promotes collagen deposition and persistent myofibroblast activation [64]. IL-6 and TNF-α act together to overstimulate fibroblasts and can down-regulate connexins, thereby enhancing fibrosis [64,65]. In advanced heart failure, elevated IL-6 levels have been correlated with symptom severity, suggesting a role in the pathogenesis of remodeling. IL-6 may contribute to cardiomyocyte hypertrophy and diastolic dysfunction in addition to fibrosis: mouse models have shown that IL-6 overexpression leads to diffuse interstitial fibrosis and increased ventricular stiffness [66]. Overall, IL-6 plays an important role in cardiac fibrogenesis as a mediator linking inflammation to fibroblasts. Chronic systemic inflammation with persistently elevated IL-6, as seen in metabolic syndrome and obesity, can, over time, lead to diffuse myocardial fibrosis and heart failure with preserved ejection fraction through its profibrotic and prohypertrophic effects [67].

##### Galectin-3

Galectin-3 (Gal-3) is a soluble lectin-family protein secreted by activated macrophages and fibroblasts, with specific binding affinity for β-galactosides. Gal-3 has been described as a biomarker and mediator in cardiac fibrosis [68,69,70]. Gal-3 can bind extracellular matrix components, affecting cell signaling. In the heart, the paracrine mechanism of Gal-3 release by cardiac macrophages and fibroblasts helps drive fibrogenesis and pathophysiologic remodeling. Gal-3 stimulates fibroblast proliferation and collagen deposition directly in the myocardium… Gal-3 also promotes a profibrotic macrophage phenotype. Gal-3 enhances TGF-β signaling, further promoting fibrosis [68,71]. Gal-3 drives fibroblasts to become myofibroblasts and also inhibits their apoptosis, prolonging their activity in injured tissue. In animal models, Gal-3 overexpression causes diffuse fibrosis. Conversely, Gal-3 inhibition reduces fibrosis and improves function [72]. Fibroblasts also produce Gal-3, creating a self-reinforcing fibrotic loop [69]. 

##### Galectin-1

Galectin-1 (Gal-1) is another member of the galectin family. In contrast to Gal-3, Gal-1 has mainly immunomodulatory effects that inhibit inflammation. Gal-1 is constitutively expressed in the heart by cardiomyocytes, endothelial cells, and resident immune cells and is released in the extracellular space where it is implicated in the resolution of inflammation in the presence of chronic inflammation [73]. In mouse infarction models, Gal-1 knockout leads to much more inflammation, dilation, and fibrosis, implying that Gal-1 normally limits adverse remodeling. The lack of Gal-1 resulted in remarkable ventricular dilatation, increased dysfunction, and diffuse fibrosis, demonstrating that Gal-1, as a physiological factor, inhibits these adverse features by reducing post-infarction inflammation and driving well-controlled tissue repair [74]. Gal-1 can inhibit TGF-β signaling (seen in liver studies), suggesting it may similarly restrain cardiac fibroblasts [75].

#### 3.2.2. Associations Between Metabolic Syndrome and Molecular Biomarkers

Metabolic syndrome (MetS) is defined as a state of low-grade chronic inflammation, evidenced by elevated serum levels of pro-inflammatory cytokines like tumor necrosis factor alpha (TNF-α) and interleukin-6 (IL-6) [76].

##### Tumor Necrosis Factor-α and Interleukin 6

As evidenced by many clinical studies, patients with MetS exhibit significantly higher concentrations of TNF-α and IL-6 than subjects without MetS, confirming that inflammation has a role in the pathogenesis of this syndrome. Excess visceral adipose tissue is infiltrated by macrophages, which secrete TNF-α and IL-6, which serve to enhance insulin resistance by modulating the signaling of insulin through peripheral tissues, thus sustaining a pro-diabetogenic vicious circle. In addition to this, TNF-α activates nuclear factor-κB (NF-κB) pathways, leading to a more aggressive inflammatory response and the release of reactive oxygen species, while IL-6 enhances hepatic C-reactive protein synthesis. Chronic inflammation from obesity is closely related to oxidative stress: oxidative stress in adipocytes has been found to promote insulin resistance and enhance local release of IL-6 and TNF-α that aggravate systemic inflammation [30,76,77]. These mediators not only mediate the degree of metabolic derangements but are also involved in cardiovascular complications. In patients with cardiometabolic risk factors, high IL-6 and TNF-α levels are related to endothelial dysfunction and progression of atherosclerosis, resulting in an increased overall risk for cardiovascular disease. Prospective meta-analyses have shown a direct association between risk of coronary events and baseline IL-6 concentrations, implying that these cytokines might be a potential biomarker of cardiovascular risk in early MetS patients [76,78].

##### Transforming Growth Factor β1

In metabolic syndrome, TGF-β1 serum levels, like classical inflammatory markers, appear elevated. For instance, those with MetS who are hypertensive tend to show markedly higher TGF-β1 concentrations than hypertensive patients without MetS [79]. Experimental data suggest that visceral obesity and insulin resistance result in TGF-β1 overexpression in adipose tissue [80]. This overactivation of the TGF-β1 axis may be the causal mechanism between metabolic state and organ damage: TGF-β1 promotes extracellular matrix deposition and fibroblast proliferation, which in turn leads to tissue fibrosis. In the context of blood vessel tissues, TGF-β1 promotes the enhancement of matrix accumulation by promoting collagen production and inhibiting its breakdown [81]. Furthermore, TGF-β1 has a role in direct vascular fibrosis from a number of cardiovascular insults, which are associated with MetS, including hypertension, hyperglycemia, and advanced glycation end-products [82]. At the cardiological level, a chronic TGF-β1–Smad pathway is maintained, favoring myocardial remodeling and mediating diastolic dysfunction and heart failure with preserved ejection fraction, features commonly observed in obese and diabetic individuals. Also, the importance of TGF-β1 as a pathogenic factor in MetS is confirmed by prognostic observations indicating that elevated levels of serum TGF-β1 were associated with new type 2 diabetes risk, irrespective of other metabolic risk factors in a population-based trial. Consequently, TGF-β1 represents another indicator of systemic fibrotic stress elicited by MetS, having both diagnostic and therapeutic significance [79,81,83].

##### Galectin-3 and Galectin-1

Galectin-3 itself is a candidate biomarker for interest in cardiometabolic diseases [84,85]. Galectin-3 levels and secretion have been significantly elevated in metabolic diseases as compared to healthy subjects. Galectin-3 is of high clinical relevance in patients with MetS. MetS was previously linked to high plasma galectin-3 levels, and the combination of MetS with galectin-3 above the 75th percentile significantly increases heart failure risks more than four-fold (over a four-fold higher long-term risk to develop heart failure) [86]. Galectin-3 has also emerged as an indicator of atrial remodeling and AF risk in cardiometabolic patients. In MetS patients who also had atrial fibrillation, significantly higher serum concentrations of galectin-3 were observed than in MetS patients without AF, leading researchers to suggest galectin-3 as a screening and prognostic marker for AF [85].

Galectin-1 is newly emerging as a potential biomarker associated with metabolic diseases. Once upon a time, galectin-1 was associated with immune modulation, but recent studies have shown that it plays a more multifaceted role in MetS. Studies with similar populations reveal increased levels of galectin-1 in obese and type 2 diabetes populations, which correlate positively with body mass index and hyperinsulinemia. In a cohort of approximately 1000 middle-aged subjects observed in the population, serum galectin-1 concentrations independently predicted obesity and insulin resistance, indicating that galectin-1 represents the level of adipose dysfunction and metabolic dysregulation. Experimental proof of concept of galectin-1 promoting adipose tissue accumulation and Pro-Inflammatory macrophage programming is performed using experimental model mice. It has been reported that galectin-1 binds to the peroxisome proliferator-activated receptor-γ (PPAR-γ) pathway, promoting weight gain and glucose intolerance associated with higher-fat diets in animals. This observation also indicates that galectin-1 could contribute to chronic low-grade inflammation and increased insulin resistance in a hypercaloric diet and metabolic inflammation [12].

### 3.3. Molecular Pathways Linking Fibrotic Biomarkers to Atrial Fibrosis

#### 3.3.1. Transforming Growth Factor β1 in Atrial Fibrosis

TGF-β1 is possibly the most prominent profibrotic mediator, also at the atrial level. For example, in chronic AF, atria do show upregulation of the TGF-β pathway as compared to sinus rhythm: in surgical patients with persistent AF, atrial tissue, TGF-β1 mRNA, and protein expression demonstrate large increases with respect to patients with sinus rhythm and have a positive correlation/effect on myocardial collagen content. Gramley et al.found that, with chronic AF prolonged from paroxysmal AF < 6 months to permanent AF > 5 years, the extent of atrial fibrosis nearly doubles compared to baseline, and there is an increase in levels of atrial TGF-β1 and Smad2/4 activation with increased duration of chronic AF in the early phase of AF. Interestingly, for very late AF (permanent AF > 5 years), TGF-β type I receptor decreased, and Smad7 increased, indicating that the longer the time after onset of AF, the lower the atrium’s response to TGF-β is over time. This biphasic effect implies that TGF-β1 underlies fibrogenesis toward the beginning of the atrial remodeling process, while additional fibrotic mechanisms are mobilized during the disease course of long-term AF [15]. A recently published study in patients with surgical Maze for AF showed a strong relationship between preoperative plasma TGF-β1 and the histologically assessed grade of left atrial wall fibrosis observed. Additionally, severe atrial fibrosis was correlated with recurrence of AF following Maze surgery and lack of recovery of atrial mechanical function after the operation [86]. Although circulating TGF-β1 is not routinely measured, a clinical trial showed that persons with persistent AF undergoing catheter ablation had markedly dilated atria in those with higher plasma TGF-β1 levels. In fact, TGF-β1 and left atrial diameter function independently and predict failure to maintain sinus rhythm after ablation. Adding TGF-β1 to a model that accounts only for atrial size greatly helps in the prediction of future AF recurrence. Patients with persistent AF having high levels of TGF-β1 are those who experience the greatest frequency of arrhythmia recurrence after ablation; the rate increases when TGF-β1 is added to the model. TGF-β1 seems to be particularly important in more severe forms of AF: in paroxysmal AF, there are no differences in cytokine levels between those who do or do not recur, while in persistent AF, the difference is clear [86,87]. TSP-1 promotes the cleavage of TGF-β1 into its active form by attaching to the latent TGF-β complex (LAP), thereby promoting cytokine-mediated activation of the fibrotic cascade. In AF atrial fibrosis, TSP-1 is significantly upregulated, supporting its pathologic importance: plasma levels of TSP-1 are elevated in atrial myocardium after AF, and plasma TSP-1 concentrations are positively related to the degree of atrial fibrosis and left atrial dilatation. Molecularly, the excessive expression of TSP-1 activates the TGF-β1/Smad3 signaling cascades of atrial fibroblasts, promoting cell division and fibroblast interstitial collagen formation. Thus, the inhibition of TSP-1 may prevent atrial fibrogenesis: for instance, inhibition of the TSP-1 and TGF-β1 complex association between TSP-1 and LSKL peptide reduced myocardial fibrosis and enhanced cardiac performance in a preclinical model [88,89,90].

#### 3.3.2. Tumor Necrosis Factor α and Atrial Inflammation

Serum TNF-α for patients with AF has been shown to be drastically increased compared to sinus rhythm subjects, controlling for cardiovascular risk factors. Furthermore, TNF-α levels seem to be elevated with persistence of AF: mean TNF-α levels are higher in people with persistent or permanent AF than paroxysmal AF. Patients with chronic AF have been shown to have circulating TNF-α concentrations significantly higher than patients with short paroxysmal episodes, which associates chronic inflammation with a persistent-state of fibrotic substrate that perpetuates the ECG condition [91]. Similarly to the ventricular myocardium, TNF-α mediates atrial fibrosis through fibroblastic activation, collagen secretion stimulation, and alterations of normal atrial architecture. In animal models, TNF-α activation causes atrial inflammation with involvement of macrophage infiltrates and interstitial collagen deposits, as well as decreased expression of connexin-40, favoring conduction dysfunction and susceptibility to AF [58]. TNF-α is present in rich amounts in the epicardial adipose tissue close to the atria. Recent studies elucidate that a large epicardial fat volume is related to extensive atrial fibrosis, which can be accounted for in part by direct secretion of pro-inflammatory cytokines into the atrial wall [58,91]. Patients displaying increased levels of TNF-α on echocardiography typically displayed left atrial dilatation and atrial dysfunction, which are processes initiated before AF [56]. More interestingly, it had also been noted that patients who experienced reduced levels of TNF-α right after ablation had increased recurrence risk, indicating that decreased levels of TNF-α might indicate an overall exhaustion of the inflammatory response that is necessary for adequate healing of ablation lesions, which requires controlled ablation. However, meta-analyses reveal overall associations of inflammatory markers with AF recurrence and that pre-ablation TNF-α is modestly but significantly higher for patients with an AF relapse after the procedure than those who remain persistently in sinus rhythm. Furthermore, nonspecific anti-inflammatory therapy has been successful in preventing postoperative AF and has been associated with reductions in AF incidence with the administration of corticosteroids after cardiac surgery [56,92].

#### 3.3.3. Interleukin-6 and Atrial Fibrosis

IL-6, an inflammatory factor, has been demonstrated to be highly associated with AF initiation and maintenance. Like TNF-α, higher levels of IL-6 are found in AF patients compared to the general population (individuals without overt structural heart disease). Additionally, as per prospective studies, IL-6 has been found to be predictive of incident AF: at-risk subgroups of patients with a higher plasma IL-6 had an increased rate of AF at follow-up [93,94]. Systemic inflammation, with IL-6 being the main marker, promotes atrial fibrogenesis by several mechanisms: IL-6 stimulates cardiac fibroblasts to synthesize collagen and express smooth muscle α-actin, leading to atrial matrix stiffening and impaired conduction homogeneity [58]. IL-6 also induces the production of CRP from hepatocytes, and CRP has been associated with the risk for the establishment of atrial thrombus and the progression of AF, indicating a global inflammatory pathway. A prospective cardiac MRI study demonstrated that AF patients with high IL-6 expression are at risk of atrial fibrosis on LGE imaging. In a multivariable analysis of the DECAAF cohort, IL-6 was independently related to the degree of fibrosis at the time of ablation, supporting the finding that patients with systemic inflammation have more pronounced fibrotic substrate. From an echocardiographic standpoint, chronically high IL-6 levels are related to left atrial enlargement and impaired atrial function. Serum IL-6 is found to correlate inversely with left atrial strain in patients with persistent AF and heart failure. The most well-characterized inflammatory biomarker to predict AF recurrence has probably been IL-6, for example. Previous research has shown that pre-ablation IL-6 levels were significantly higher in patients with AF recurrence after ablation than in sinus rhythm patients. The finding implies that the presence of an active inflammatory status contributes to an incomplete or suboptimal ablation site healing and maintenance of an arrhythmogenic substrate. In practice, combining IL-6 with other markers enhances the prediction of AF recurrence post-ablation [94,95]. Additionally, treatments that reduce IL-6 could lower AF risk: in the CANTOS trial, a secondary analysis found a reduction in hospitalizations for AF in the treated group, which may indicate a benefit of attenuating the inflammatory cascade on atrial arrhythmogenesis [95].

#### 3.3.4. Galectin-3 in Atrial Fibrosis

Galectin-3 is also directly involved in cardiac fibrogenesis. AF is associated with a much higher serum Gal-3 level compared to sinus rhythm in a number of studies. Clementy et al. found that the median Gal-3 in non-valvular AF patients was approximately twice that of age-matched controls. Second, Gal-3 is linked to AF type: persistent AF is associated with higher Gal-3 levels for patients than paroxysmal AF, indicative of a subsequent evolution of fibrosis and inflammation with the chronicity of the arrhythmia [16]. In 76 patients, mean Gal-3 levels in persistent AF were 17.8 ng/mL, in paroxysmal AF were 13.4 ng/mL, and control levels were ~9 ng/mL, once again confirming high specificity of the biomarker to AF phenotype [96]. Serum Gal-3 correlates with the extent of non-invasive assessment of atrial fibrosis. Pre-procedural LGE-MRI quantification of atrial fibrosis was carried out in 33 patients with paroxysmal AF with cryoballoon ablation. Serum Gal-3 levels were found to correlate independently and positively with the percentage of atrial fibrosis on MRI, even after adjusting for atrial volume. Gal-3 also strongly correlated with atrial electromechanical delay (AEMD); greater Gal-3 values were associated with prolonged AEMD, with elevated values indicating inhibited atrial conduction presumably because of diffuse fibrosis. A significant value of CEI is left atrial volume index (LAVi). In the same study, LAVi and Gal-3 were both independent predictors of atrial fibrosis extent among paroxysmal AF patients [97]. The association between Gal-3 and atrial function was also demonstrated in a further study of patients with persistent AF and dilated atria, revealing an inverse correlation in this disease [98]. Galectin-3 is a well-characterized biomarker for AF recurrence following therapy. In regard to catheter ablation, serum Gal-3 has consistently been found to predict a higher risk of AF relapses when pre-procedurally elevated. In a cohort with 50 patients with persistent AF, median Gal-3 levels demonstrated increased levels in these patients who went on to recur after ablation, with the independent predictor of recurrence being Gal-3 [99]. Another similar study of 160 patients also showed that only serum Gal-3 and left atrial diameter were independent predictors of recurrence at 12 months postablation. A Gal-3 threshold ≥15 ng/mL was suggested by the authors, which, in conjunction with left atrial diameter ≥40 mm, identified a subgroup that showed a very high recurrence rate (≈59%) as opposed to a ≈9% recurrence in those below both thresholds [100]. Gal-3 has implications, especially for an electrical cardioversion program: a Gal-3 > 17 ng/mL was correlated with a two-fold increased risk of early recurrence after cardioversion in patients with persistent AF. In cohorts of ≈100 patients, a finding of an association not found is consistent with previous studies linking Gal-3 to ablation outcome, as Gal-3 may reflect chronic fibrotic status in some patients rather than an acute effect from ablation. However, a recent meta-analysis supports that patients have significantly higher pre-ablation Gal-3 in recurrence patients, and it also recommends incorporating Gal-3 in risk scores of AF recurrence. In addition to recurrence, galectin-3 was also found to be associated with the progression of AF: in a longitudinal study of patients with paroxysmal AF, patients with progression into persistent AF had significantly higher baseline Gal-3 than patients who remained paroxysmal [16]. In AF patients, another feature is that Gal-3 has a direct correlation with thrombus formation in the left atrial appendage, which can be attributed to its association with atrial fibrosis, stasis, and reduced appendage emptying [97]. Galectin-3 is a biomarker of atrial fibrosis, as its levels determine the degree of fibrosis and atrial remodeling and are associated with early detection of AF patients with a high rate of recurrence after ablation or progression to permanent forms. Integrating Gal-3 with other risk factors in clinical risk-score estimation is demonstrated to improve the accuracy in AF recurrence prediction [101].

#### 3.3.5. Galectin-1 and Atrial Remodeling

In contrast to galectin-3, galectin-1 provides its own beneficial role in atrial fibrosis as well as AF, although the latter is less well defined than the former. Considering the anti-inflammatory effects, Gal-1 is expected to protect against excessive atrial fibrosis by limiting local inflammation that leads to fibroblast activation. The association of Gal-1 with more orderly structural healing and decreased ventricular dilatation in experimental models of myocardial infarction may be beneficial, as Gal-1 opposes profibrotic mechanisms and slows substrate formation. An intriguing clinical clue seems to be the positive correlation of Gal-1 level with atrial size: in patients with heart failure, more Gal-1 in serum correlates with left atrial dilatation [18]. Despite resisting its initial hypothesis, this correlation suggests Gal-1 increases compensatory pathways in cases of pronounced atrial overload and advanced fibrosis in order to alleviate the chronic inflammation that accompanies the dilated atrium. As such, an enlarged atrium could induce Gal-1 release from atrial cardiomyocytes and immune cells in order to mitigate the subsequent development of fibroblasts that result from them. There are currently no large population-based clinical trials that have quantitatively reported galectin-1 in AF patients or investigated the association of galectin-1 with atrial fibrosis or post-ablation recurrence. Nevertheless, indirect data from postoperative AF have indicated that AF develops after cardiac surgery in patients with reduced preoperative Gal-1 levels compared with patients without, leading investigators to speculate that relatively low levels of Gal-1 may predispose patients to an uncontrolled inflammatory–fibrotic response. It is unknown if circulating Gal-1 could influence the evolution of paroxysmal to persistent AF. Due to its strong association with inflammation, one would expect that low Gal-1 levels would contribute to excessive risk of uncontrolled fibrotic remodeling and progression to permanent AF, but high Gal-1 levels would help maintain and prolong fibrotic normalization, leading to a continuation of sinus rhythm. However, these hypotheses need to be formally validated.

## 4. Conclusions

In summary, the pro-inflammatory cytokines IL-6 and TNF-α, the profibrotic growth factor TGF-β1, and the lectins galectin-3 and galectin-1 contribute to the construction of a common pathophysiological axis through that of metabolic syndrome, chronic inflammation, myocardial fibrosis, and atrial arrhythmogenic substrates. Increased numbers of these biomarkers in metabolic syndrome patients suggest an environment conducive to cardiac remodeling. The strong association indicates that systemic inflammation induced by MetS causes fibroblast activation via mediators such as TGF-β1, leading to an increase in the production of myocardial collagen tissue in the latter half of this time and chronic in the latter half. Galectin-3, mainly synthesized by macrophages, acts between inflammation and fibrosis to activate profibrotic TGF-β/Smad pathways as well as induce structural atrial remodeling. Simultaneously, galectin-1 has a twofold, context-dependent effect, such that a transient elevation may lead to resolution of inflammation and tissue repair, while prolonged, excess Gal-1 overproduction promotes fibrosis by stimulating fibroblasts and excessive extracellular matrix accumulation. Therefore, the aggregated biomarker set illustrates the extent of inflammation and the burden of cardiac fibrosis, revealing which shared mechanisms relate to MetS and atrial fibrillation. The clinical relevance of these markers stems from their ability to enhance cardiovascular risk categorization and modulate specific therapy. In contrast, in the setting of MetS, a pro-inflammatory/profibrotic profile could assist in identifying patients at risk for elevated cardiovascular and arrhythmic consequences, for which an aggressive risk factor control would be justified. In proven atrial fibrillation, circulating levels of Gal-3 and TGF-β1 have been reported as independent predictors of AF recurrence following catheter ablation, suggesting that a patient with AF and with significantly raised levels of these markers probably contains an extensive fibrotic substrate and a higher risk for relapses. In addition, biomarker-based clinical risk score incorporation has already demonstrated incremental benefit. Likewise, recognition of patients with marked systemic inflammation could inform refinement of approaches to avoid complications. We note that, as a narrative, the selection of studies is subject to potential selection and publication bias. This approach does not follow the formal methodology of a systematic review, so some relevant literature may not have been captured. Nonetheless, our goal was to provide a focused synthesis of key findings. Future research will evaluate these biomarkers as a screening tool and therapeutic target in clinical practice. The view here is that integrated clinical–biological scores are especially promising in those with MetS who exhibit tendencies towards developing AF and in other patients with MetS who have AF who might benefit from tailored medical strategies that are tailored according to their biomarker profile.

## Figures and Tables

**Table 1:** Summary of key inflammatory and fibrotic biomarkers implicated in myocardium remodeling.

Biomarker	Primary Signaling Pathway	Proposed Role in Atrial Fibrillation
TGF-β1	Smad-dependent TGF-β signaling	Master profibrotic cytokine; promotes fibroblast-to-myofibroblast differentiation, collagen deposition, and cardiac structural remodeling.
TNF-α	TNF-α/NF-κB signaling	Potent pro-inflammatory mediator; stimulates fibroblast proliferation, extracellular matrix deposition, and arrhythmogenic electrical remodeling in the myocardium.
IL-6	IL-6/JAK/STAT3 signaling	pro-inflammatory cytokine; activates STAT3 in fibroblasts to induce collagen production and contribute to fibrosis and remodeling.
Galectin-3	Gal-3/TGF-β—Smad signaling	Profibrotic lectin promotes fibroblast activation to myofibroblast transformation and collagen synthesis, amplifying TGF-β-driven fibrosis and adverse remodeling.
Galectin-1	Gal-1-mediated immunomodulatory signaling	Immunoregulatory lectin promotes resolution of inflammation, inhibits fibroblast activation, and may attenuate atrial fibrosis.

## Data Availability

No new data were created or analyzed in this study. Data sharing is not applicable to this article.

## Abbreviations

The following abbreviations are used in this manuscript:

AF	Atrial fibrillation
AS	Left atrium
AD	Right atrium
LAVI	Left atrial volume index
LASr	Left atrial reservoir strain
AEMD	Atrial electromechanical delay
MetS	Metabolic syndrome
HFpEF	Heart failure with preserved ejection fraction
HF	Heart failure
TGF-β1	Transforming Growth Factor beta-1
TNF-α	Tumor necrosis factor alpha
IL-6	Interleukin-6
Gal-3	Galectin-3
Gal-1	Galectin-1
CRP	C-reactive protein
MMP	Matrix Metalloproteinases
ROS	Reactive oxygen species
TSP-1	Thrombospondin-1
LAP	Latency Associated Peptide
ECM	Extracellular matrix
PPAR-γ	Peroxisome Proliferator-Activated Receptor gamma
Smad2/3/4/7	SMAD proteins involved in TGF-β signaling
JAK/STAT3	Janus Kinase/Signal Transducer and Activator of Transcription 3
NF-κB	Nuclear Factor kappa-B
LGE-MRI	Late Gadolinium Enhancement Magnetic Resonance Imaging
MRI	Magnetic Resonance Imaging
TTE	Transthoracic echocardiography
ECG	Electrocardiogram
RAAS	Renin–Angiotensin–Aldosterone System
ACEis	Angiotensin-Converting Enzyme inhibitors
ARBs	Angiotensin Receptor Blockers
EAT	Epicardial adipose tissue
DECAAF	Delayed Enhancement MRI Determinant of Atrial Fibrillation
RR	Risk Ratio
HR	Hazard Ratio
